# A Case of Persistent Human Pegivirus Infection in Two Separate Pregnancies of a Woman

**DOI:** 10.3390/microorganisms10101925

**Published:** 2022-09-28

**Authors:** Mathieu Garand, Susie S. Y. Huang, Lisa S. Goessling, Donna A. Santillan, Mark K. Santillan, Anoop Brar, Todd N. Wylie, Kristine M. Wylie, Pirooz Eghtesady

**Affiliations:** 1 Division of Pediatric Cardiothoracic Surgery, Department of Surgery, Washington University School of Medicine, St. Louis, MO 63110, USA; 2 Department of Obstetrics and Gynecology, University of Iowa, Iowa City, IA 52242, USA; 3 Department of Pediatrics, Washington University School of Medicine, St. Louis, MO 63110, USA; 4 McDonnell Genome Institute, Washington University School of Medicine, St. Louis, MO 63110, USA

**Keywords:** prenatal infection, virome, viral antibody, VirScan, ViroCap, maternal viral infection, viral protein, GBV-C, placenta, fetal viral infection

## Abstract

Human pegivirus (HPgV) is best known for persistent, presumably non-pathogenic, infection and a propensity to co-infect with human immunodeficiency virus or hepatitis C virus. However, unique attributes, such as the increased risk of malignancy or immune modulation, have been recently recognized for HPgV. We have identified a unique case of a woman with high levels HPgV infection in two pregnancies, which occurred 4 years apart and without evidence of human immunodeficiency virus or hepatitis C virus infection. The second pregnancy was complicated by congenital heart disease. A high level of HPgV infection was detected in the maternal blood from different trimesters by RT-PCR and identified as HPgV type 1 genotype 2 in both pregnancies. In the second pregnancy, the decidua and intervillous tissue of the placenta were positive for HPgV by PCR but not the chorion or cord blood (from both pregnancies), suggesting no vertical transmission despite high levels of viremia. The HPgV genome sequence was remarkably conserved over the 4 years. Using VirScan, sera antibodies for HPgV were detected in the first trimester of both pregnancies. We observed the same anti-HPgV antibodies against the non-structural NS5 protein in both pregnancies, suggesting a similar non-E2 protein humoral immune response over time. To the best of our knowledge, this is the first report of persistent HPgV infection involving placental tissues with no clear indication of vertical transmission. Our results reveal a more elaborate viral-host interaction than previously reported, expand our knowledge about tropism, and opens avenues for exploring the replication sites of this virus.

## 1. Introduction

Human pegivirus (HPgV) is a member of the *Pegivirus* genus within the *Flaviviridae* family [1]. It is an enveloped virus, containing a positive-sense, single-stranded RNA genome of ~9500 nucleotide, like that of hepatitis C virus (HCV), another member of the *Flaviviridae* family. HPgV has two structural proteins (E1 and E2), two predicted proteins of undetermined function (X and p* protein), and six non-structural (NS) proteins [1,2,3,4]. To date, seven genotypes of HPgV-1 (species *Pegivirus C*, formerly GBV-C) have been classified, along with many variants [1,5,6], which vary in their distributions across the globe.

Knowledge regarding the biology and behavior of this virus remains scarce. HPgV-1 is a lymphotropic virus with a positive association between viremia with a risk of adult lymphomas [7,8]. The prevalence of HPgV-1 infection in developed countries ranges from 0.5 to 5% [9,10]. Transmission of HPgV-1 primarily occurs through exposure to infected blood or sexual contact, and has been documented among intravenous drug users [11], blood transfusions recipients [3], patients with parenteral exposure [12], and transplant recipients [12,13,14]. After HPgV-1 infection, 20–30% of people develop chronic infections [15]. Viremia is typically cleared within 2 years in the majority of immune competent individuals [16,17]. Antibodies directed toward the envelope HPgV glycoprotein E2, which is thought to be the immunodominant antigenic site, are detected as viremia is cleared [18]. While the pathogenicity of this virus remains unclear, HPgV-1 has a propensity to co-infect individuals with other viral infections, particularly human immunodeficiency virus (HIV) [19,20] and HCV [6,21]. HPgV interferes with the pathogenicity of HIV [22] and slows disease progression [7,9,19,23,24]. Indeed, epidemiological evidence showed that longer period of HPgV viremia is associated with greater survival among HIV-positive individuals [7]. The effect is mediated by a reduction in HIV replication within HPgV-infected CD4 T cells (compared to non-HPgV infected) [23] and recent studies demonstrating HIV entry interference by HPgV E2-related peptides [22].

In pregnant women, the prevalence of HPgV infection is reported to be 1.1–6%, with a presumed rate of vertical transmission of up to ~65%, without HCV or HIV co-infection. These reports, however, were all from small cohorts and the route of transmission was not determined and only presumed to have been trans-placental based on detection of the virus in the newborn infant (8 positives out of 12 children, or 66.7%, born from HPgV positive mothers [25]; and 13 positives out of 36 children, or 65%, born from HPgV positive mothers [26]). This presumed mother-to-infant transmission rate exceeds that of other viruses; for examples the rate of transmission for HIV is 23% [27], and the rate of transmission for HCV in mothers co-infected with HIV is 36% [28]. HPgV infection in infants can persists up to 12 months with no reported adverse health effects [29]. To the best of our knowledge, no one has clearly demonstrated vertical transmission by testing maternal and fetal placental tissues, and umbilical cord blood.

Here, we present HPgV infection in a woman, spanning four years and two pregnancies, with no clear indication of vertical transmission. We used two comprehensive technologies to characterize the infection: (1) ViroCap to detect and genotype the virus [30,31], and (2) VirScan, to profile anti-viral IgG responses at epitope resolution [32]. We then tested for the presence of HPgV nucleic acids by end-point PCR in first-trimester maternal plasma and umbilical cord blood samples from both pregnancies as well as maternal and fetal placental specimens from the second pregnancy.

## 2. Materials and Methods

### 2.1. Study Samples

**Figure 1:**
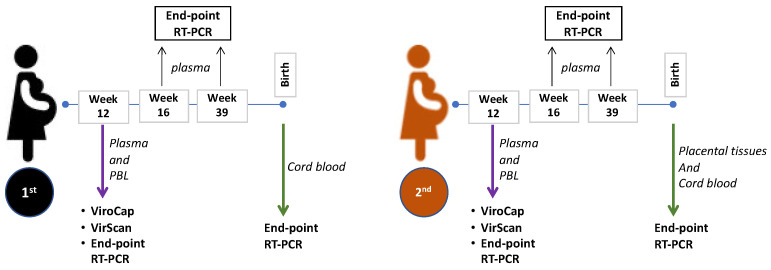
Summary of sample collection and assays. First trimester plasma and peripheral blood leukocytes (PBL) were collected/isolated (week 12 and 11 for 1st and 2nd pregnancy, respectively). ViroCap and VirScan were performed on the plasma while end-point RT-PCR was performed on plasma and PBL. Additional samples collected during the 2nd and 3rd trimesters (as indicated in weeks) were assayed with RT-PCR. At birth, placental tissues (only 2nd pregnancy) and cord blood were collected for RT-PCR assays.

We obtained samples from pregnant women who provided informed consent for the collection of biological samples and clinical data (Washington University in St. Louis; IRB#202002043; University of Iowa Maternal Fetal Tissue Biobank; IRB#200910784). ViroCap, VirScan and RT-PCR assays were performed on 1st-trimester plasma samples corresponding to week 12 and 11 for the first and second pregnancies, respectively. Additional plasma samples were collected at week 16 and 39 for both pregnancies, as well as cord blood at both births and the placental/fetal tissues at post-partum for the second pregnancy, were used for end-point RT-PCR assays as shown in Figure 1.

### 2.2. Pegivirus Sequence Analysis

Nucleic acid isolated from samples with Quick DNA/RNA Viral Kit (Zymo Research, # D7020) was used was used as input for the ViroCap assay [31]. Viral sequences were analyzed with the ViroMatch pipeline [33]. After identifying HPgV sequences in the two maternal samples, HPgV genomes were assembled with IDBA-UD [34] and contigs were extended with PRICE software [35]. The assemblies were manually reviewed with Tablet [36]. Genomes were compared with NCBI BLAST [37] and MUSCLE [38] programs. For genotyping, phylogenetic trees were constructed using the NIAID Virus Pathogen Database and Analysis Resource online through the web site at www.viprbrc.org (last accessed 7 January 2022) [39]. Phylogeny was estimated using the maximum likelihood method with RAXML with 100 bootstraps and annotated with iTOL [40].

### 2.3. RT-PCR Validation

The presence of HPgV was analyzed by nested-PCR [41] and one-step RT-qPCR [42] from the extracted nucleic acid. The HPgV RNA quantitation standard used was kindly provided by Dr. Jack Stapleton. For the nested-PCR reactions, primer sequences for PCR1 were as follows: HGV1 forward 5’-AGGTGGTGGATGGGTGAT-3′; HGV2 reverse 5′-TGCCACCCGCCCTCACCCGAA-3′. Primer sequences for PCR2 were as follows: HGV3 forward 5′-TGGTAGGTCGTAAATCCCGGT-3′; HGV4 reverse 5′-GGAGCTGGGTGGCCCCATGCAT-3′.

### 2.4. Viral Antibody Analysis

VirScan assay was performed as previously described [43]. In brief, 2 µg of plasma IgG were added to phage input library at 1.3 × 10^10^ pfu for complex formation at 4 °C overnight. Immunoprecipitation (IP) was then performed at 4 °C for 4 h using 1:1 ratio of Protein A and Protein G Dynabeads mix (Invitrogen). As described previously [43], beads were collected by centrifugation, washed twice with 300 µL of the PhIP-seq wash buffer (50 mM Tris-HCl, 150 mM NaCl, 01% NP-40 at pH 7.5), and resuspended in 19 µL of 1X PCR 1 master mix. Two rounds of PCR were performed to amplify the phage DNA and introduce sample specific barcodes and sequencing adapters prior to sequencing on a NextSeq 500 (Illumina).

Sequences were aligned to the Vir3 reference genome (obtained from Dr. Steve Elledge) using Bowtie2 (version 2.2.6) with the option –very-sensitive-local. Samtools (version 1.6) was used to index and sort the BAM files and raw counts generated using the option -idxstats. All counts were normalized to 1 million reads prior to additional downstream analyses. The VirScan analysis pipeline was performed as previously described [44]. In brief, peptides from the MockIPs (controls without serum) were binned according to their rank to define the null distributions for calculating the standard unit (i.e., z score) for enrichment which is referred to Epitope Binding Signal (EBS). Significant non-specific background binding against a particular peptide is determined when a peptide has a standard unit > 3.5 for both technical replicates and in 2 or more MockIPs. The standard unit for each peptide in all samples were then calculated against the binned null distributions derived independently within each sample. A peptide with a standard unit >3.5 was deemed significantly enriched. To filter cross-reactive antibodies, we calculated the mean similarity score (sscore) by Damerau-Levenshtein distance (R package stringdist, method = ‘dl’) for each significantly enriched peptide sequence against all other significantly enriched peptides belonging to the same species and only retained those with score < 0.125. We also removed peptides that shared >7 contiguous amino acid sequence. The remaining significantly enriched peptides that satisfied all the filter were defined as “specific”. Alignment of significantly enriched HPgV peptides was performed with UniProt Align tools (/www.uniprot.org/align/; accessed on 15 September 2022) to determine each peptide’s starting and ending position. Then, we manually aligned the peptides with the polyprotein sequence.

## 3. Results

### 3.1. Maternal Medical History

The patient is a Caucasian woman (not Hispanic or Latino) living in the United States of America at the time of recruitment and sample collection. The first pregnancy (at maternal age of 30 years) of the individual resulted in a normal spontaneous vaginal delivery and the infant and postpartum course were normal. The second pregnancy 4 years later (at maternal age of 34 years) was complicated by congenital heart disease (CHD) (multiple muscular ventricular septal defects). Maternal body-mass index was 20.4 and 21.76 for the first and second pregnancies, respectively. Prior and during both pregnancies, the mother had no history of gestational diabetes, hypoglycemia, hyperglycemia, alcohol use, or tobacco use. The newborn of the first pregnancy had a gestational age of 39 weeks and 5 days, a birth weight of 3972 g, and is a male. The second pregnancy’s newborn had a gestational age of 39 weeks, a birth weight of 3434 g, and is a female. The mother had a history of human papillomavirus (HPV) infection. Prior to her second pregnancy, the mother developed an eye melanoma. She also had 3 liver lesions (hemangioma/benign cysts) during her second pregnancy which was also complicated by iron-deficiency anemia. The mother was negative for HIV and hepatitis B surface antigen by serology testing in the clinic. There are no other covariates known to affect HPgV infection. A summary of the samples obtained, and assays performed is shown in Figure 1.

### 3.2. Viral Sequence Analysis

**Figure 2:**
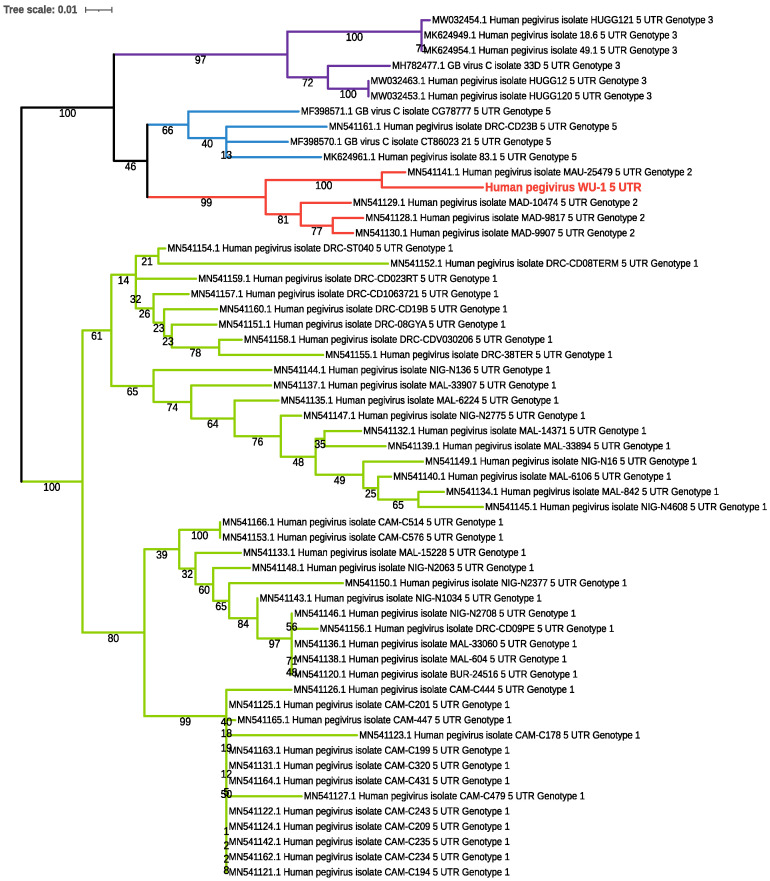
The human pegivirus sequenced in the first trimester maternal plasma samples shows high homology to HPgV-1 genotype 2. The 5′ UTR of the newly sequenced pegivirus genomes were compared to publicly available, curated genotyped 5′ UTR sequences (MN541120-MN541174) and supplemented with and additional sequences from GenBank to add genotype representation (MW032463.1, MW032454.1, MW032453.1, MK624961.1, MK624954.1, MK624949.1, MF398571.1, MF398570.1, and MH782477.1). Clades representing genotypes 1 (green), 2 (red), 3 (purple), and 5 (blue) are shown. The sample from this study is in the genotype 2 clade and is labeled in red font. Bootstrap values are shown on each branch.

By viral sequence analysis of first trimester maternal plasma from both pregnancies, we identified the complete coding sequences of the HPgV genomes. The consensus sequences from both genomes were identical and without mutations. The sequence was ~91% identical to the best match in the NCBI nucleotide database (Sequence ID MN551063.1), and the virus was determined to be most similar to HPgV-1 genotype 2 based on comparison with representative 5′-untranslated region (UTR) sequences from known genotypes (Figure 2).

### 3.3. VirScan Analysis

**Figure 3:**
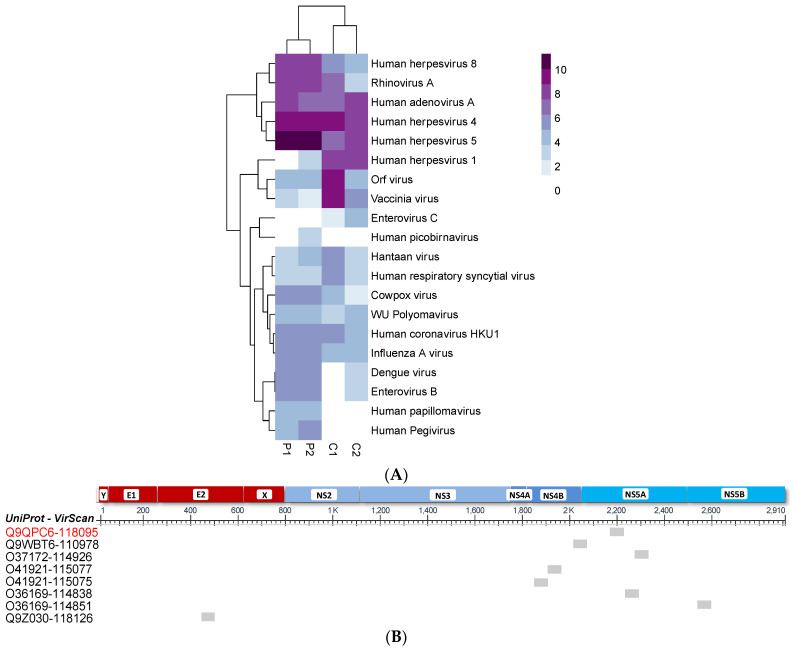
VirScan identified enriched anti-viral antibodies. (**A**) Heatmap with hierarchical clustering of the cumulative sum of significant peptide Epitope Binding Signal (EBS) in log_2_ (gradient, 1.8 to 10) of each virus species in the two pregnancies and two unrelated healthy adult volunteers (Control, C). Absence of antibody is indicated in white. P1: First pregnancy; P2: Second pregnancy. (**B**) Alignment positions of the peptide that passed through all our filtering (red text) and the other peptides (black text) are denoted by grey boxes. The row labels indicate the identifier from the VirScan library and consist of a UniProt entry ID and a VirScan peptide ID. Each peptide is aligned to the different portion of the polyprotein represented at the top of the figure by red and blue boxes, and the amino acid position is denoted just below.

Using VirScan, we observed evidence of antibody responses against several viral species, including commonly observed group of viruses such as human herpesviruses, rhinoviruses, and adenoviruses, which corroborates findings from other groups [44,45]. Relevant to this study, VirScan detected the presence of antibodies to HPgV peptides in the two 1st-trimester maternal samples (Figure 3A). There were several reactive HPgV peptides significantly enriched in those samples, but one passed through all our filtering and was considered specific (entry Q9QPC6-118095, Figure 3B). The HPgV enrichment had a log_2_ EBS of 5.013 and 5.757 in the first (P1) and second (P2) pregnancies, respectively.

### 3.4. Detection of HPgV by PCR

From both pregnancies, the maternal plasma samples consistently tested positive for HPgV by end-point RT-PCR, but the cord blood samples were negative (Table 1). Taking into consideration the possibility of low copy number or cross-contamination, RT-PCR assays were repeated several times. The detection in the maternal peripheral blood leukocytes was inconsistent (positive in 2 out of 8 independent RT-PCR reactions of the same sample preparation). In the second pregnancy, the decidua and intervillous tissue of the placenta were consistently positive for HPgV (positive in 8 out of 8 independent RT-PCR reactions of the same sample preparation); however, the chorion was negative. Results from the amnion was also inconsistent and showed a faint band in 2 out of 5 independent RT-PCR reactions of the same sample preparation.

## 4. Discussion

Prolonged persistence of the HPgV have been noted [7,13,46], including in infants born to mothers positive for HPgV [25,29]. However, none have documented persistence of the virus over years and in multiple pregnancies. Our study, to our knowledge, is the first to show the presence of HPgV infection during two pregnancies from the same woman, 4 years apart. The patient had no prior molecular evidence for HIV or HCV infection. The first pregnancy resulted in a normal baby, with no detectable HPgV in the cord blood. In contrast, the second pregnancy was complicated by CHD. The decidua and intervillous tissue of the placenta from this second pregnancy were strongly positive for HPgV, while the cord blood and chorion showed no evidence of this virus. Altogether, the findings suggest the absence of vertical transmission in both pregnancies. However, we acknowledge our conclusion is limited by the absence of testing for HPgV in the newborns. The inconsistent (2/5) faint PCR bands in the amnion most likely reflects maternal tissue or blood contamination as HPgV cDNA was found to be present in high abundance in the maternal blood and placental tissues. Surprisingly, detection in maternal peripheral blood leukocytes was also inconsistent, given the reported lymphotropic nature of the virus. These data suggest low copy number of the virus in leukocytes and support the idea that we detected mostly free, circulating virus in the plasma. It is also conceivable that the endothelial cells may be an alternative site for persistence of the virus.

To put our findings into perspective, a small study from Brazil found that among 63 pregnant women under treatment for HIV, 25% had HPgV infection. Of those with HPgV, five had HPgV-positive newborns, corresponding to a vertical transmission of 31% [47]. Pre-natal care was the only significant risk factor found, suggesting that extravasation and exposure to HPgV in the vaginal mucosal as one of the routes of transmission. In this study, we did not have access to samples from the newborns at birth or from follow-up. In another study from Italy, Paternoster et al. found that among 879 healthy pregnant women enrolled, 4.1% had HPgV infection [26]. Of those women, vertical transmission to the newborn was 65% at birth. However, none of the clinical parameters reported were significantly associated with infection status, including the type of delivery. In all, the determining risk factor(s) and the main route of vertical transmission of HPgV either in HIV or healthy cohorts are still unclear.

Several viral infections have a documented history of causing birth defects, including congenital heart disease. In the 1970, immunologists created ToRCH as a group of viruses associated congenital malformations [48]. The recent years epidemic (Zika virus, COVID-19) highlights the risk associated with viral infection on fetal development [49]. We and others have previously shown that viruses can cross the placenta and damage the developing heart [50,51,52]. The second pregnancy was complicated by CHD; however, there was insufficient evidence from our data to suggest a causative effect of the persistent maternal HPgV infection. The absence transmission of the virus to the fetal placental tissues suggests direct fetal HPgV infection is unlikely to be a contributing factor in this case of CHD. We cannot, however, rule out the possibility of transient vertical transmission at an earlier point during pregnancy (e.g., 1st trimester) since we only examined the placenta at delivery.

Detection of antibodies targeting the HPgV E2 protein is associated with viral clearance. There have been no reported antibody responses to the NS proteins in HPgV-1 infections [1]. We were able to detect the presence of anti-HPgV-1 antibody responses simultaneously with evidence of viremia during both pregnancies. The notable peptide identified, from our VirScan analysis, in the mother as part of this immune response is a 56 aa fragment of the non-structural protein NS5 (EBS = 5.013 and 5.757 in the first and second pregnancies, respectively), encoded by the NS5 gene of HPgV-1. This 56-aa peptide sequence maps to amino acid positions 2142–2197 of the HPgV-1 genotype 2 polyprotein (E-value: 1.1 × 10^−38^; score = 324, UniProtKB: A0A895ZPP4_9FLAV). The molecular function of the NS5 protein includes RNA binding, and RNA-directed 5′-3′ RNA polymerase activity and is involved in viral RNA genome replication [53]. Interestingly, the peptide fragments of this NS5 protein have been proposed as serological markers for HPgV-2 infection, a new HPgV species recently discovered [54]. Perhaps the antibody profile we are seeing is a marker for those with persistent pegivirus viremia, which occurs in about 25% of those infected. Of note, a study found that amino acid polymorphisms in NS5A sequence (but not in E2 sequence) affected the sensitivity to interferon therapy and proposed this mechanism as a evasion method used by HPgV [55]. Further studies are warranted to further delineate these immune responses. Importantly, our results show the potential utility of VirScan technology where no commercial antibodies are available, as in the case with HPgV.

Several genotypes of HPgV-1 have been identified world-wide by genome sequencing [3,6,12,56,57]. HPgV-1 genotyping in our subject showed high homology to HPgV-1 genotype 2. The complete genome sequence remained remarkably unchanged over the 4-year interval between the collections of samples. Thus, the VirScan, ViroCap and PCR results appear to be concordant with a maintained pattern of infection by our HPgV-1 isolate. We are not aware of other studies that have evaluated HPgV genome sequence or antibody response over time in the same patient, so whether this lack of change is unusual for HPgV-1 is an interesting question for future studies.

In summary, we have identified a unique case of persistent HPgV-1 in a woman with two pregnancies in 4 years. Although the mother had HPV infections, the etiology of the HPgV-1 infection in our patient is not known and did not coincide with common co-infections (HIV and HCV). To the best of our knowledge, we are the first group to directly assess vertical transmission by testing fetal and placental tissues, and umbilical cord blood for the presence of HPgV. We saw no evidence of vertical transmission of the virus in two consecutive pregnancies. Our observation of this long-term persistent HPgV-1 infection and humoral response to NS5 epitopes during pregnancies highlights a complex interplay between virus and host and suggests putative effects on maternal health and fetal development to be explored in future studies.

## Figures and Tables

**Table 1:** Detection of Pegivirus (HPgV) nucleic acid by end-point PCR in maternal 1^st^-trimester plasma, peripheral blood leukocytes (PBL), decidual and intervillous tissue of the placenta (IVTP), and fetal (cord blood, amnion, and chorion) samples.

Pregnancy	Maternal Plasma	PBL	Decidua	IVTP	Cord Blood	Amnion	Chorion
1st	++	+/−			−		
2nd	++	+/−	++	++	−	+/−	−

“++” indicates strongly detected, “−“ indicates not detected, and “+/−” indicates weak and inconsistent detection. The absence of symbol indicates the tissue was not available. RT-PCR assay was repeated at least 5 times from the same sample preparation for all tissues.

## Data Availability

The data used to support the findings of this study are available from the corresponding author upon request.

## Abbreviations

CHD	congenital heart disease
HCV	hepatitis C virus
HIV	human immunodeficiency virus
HPgV	human pegivirus
HPV	human papillomavirus
IVTP	intervillous tissue of the placenta
PBL	peripheral blood leukocytes
UTR	untranslated region
